# Effects of a DXA result letter on satisfaction, quality of life, and osteoporosis knowledge: a randomized controlled trial

**DOI:** 10.1186/s12891-016-1227-0

**Published:** 2016-08-26

**Authors:** Stephanie W. Edmonds, Peter Cram, Yiyue Lou, Michael P. Jones, Douglas W. Roblin, Kenneth G. Saag, Nicole C. Wright, Fredric D. Wolinsky, On Behalf of the PAADRN Investigators, Jeffrey R. Curtis, Sarah L. Morgan, Janet A. Schlechte, David J. Zelman

**Affiliations:** 1 Carver College of Medicine, Department of Internal Medicine, University of Iowa, 5231 Westlawn, IA 52242 Iowa City, IA USA; 2 College of Nursing, University of Iowa, Iowa City, IA USA; 3 Department of Medicine, University of Toronto Division of General Internal Medicine, Toronto, ON Canada; 4 University Health Network and Mount Sinai Hospital, Toronto, ON Canada; 5 College of Public Health, Department of Biostatistics, University of Iowa, Iowa City, IA USA; 6 Iowa City Veterans Affairs Health System, Iowa City, IA USA; 7 Kaiser Permanente, Atlanta, GA USA; 8 School of Public Health, Department of Health Management and Policy, Georgia State University, Atlanta, GA USA; 9 Department of Internal Medicine, Division of Clinical Immunology and Rheumatology, University of Alabama at Birmingham, Birmingham, AL USA; 10 School of Public Health, Department of Epidemiology, University of Alabama at Birmingham, Birmingham, AL USA; 11 College of Public Health, Department of Health Management and Policy, University of Iowa, Iowa, IA USA

**Keywords:** Osteoporosis, Quality of life, Quality of health care, Health education, Dual-energy x-ray absorptiometry

## Abstract

**Background:**

Undiagnosed, or diagnosed and untreated osteoporosis (OP) increases the likelihood that falls result in hip fractures, decreased quality of life (QOL), and significant medical expenditures among older adults. We tested whether a tailored dual energy x-ray absorptiometry (DXA) test result letter and an accompanying educational bone-health brochure affected patient satisfaction, QOL, or OP knowledge.

**Methods:**

The Patient Activation after DXA Result Notification (PAADRN) study was a double-blinded, pragmatic, randomized trial which enrolled patients from 2012 to 2014. We randomized 7,749 patients presenting for DXA at three health care institutions in the United States who were ≥ 50 years old and able to understand English. Intervention patients received a tailored letter four weeks after DXA containing their results, 10-year fracture risk, and a bone-health educational brochure. Control patients received the results of their DXA per the usual practices of their providers and institutions. Satisfaction with bone health care, QOL, and OP knowledge were assessed at baseline and 12- and 52-weeks after DXA. Intention-to-treat analyses used multiple imputation for missing data and random effects regression models to adjust for clustering within providers and covariates.

**Results:**

At 12-weeks 6,728 (86.8 %) and at 52-weeks 6,103 participants (78.8 %) completed their follow-up interviews. The intervention group was more satisfied with their bone health care compared to the usual care group at both their 12- and 52-week follow-ups (standardized effect size = 0.28 at 12-weeks and 0.17 at 52-weeks, *p* < 0.001). There were no differences between the intervention and usual care groups in QOL or OP knowledge at either time point.

**Conclusions:**

A tailored DXA result letter and bone-health educational brochure sent to patients improved patient satisfaction with bone-related health care.

**Trial registration:**

Clinical Trials.gov Identifier: NCT01507662 First received: December 8, 2011.

## Background

Although only 10 % of Americans ≥ 50 years old are currently diagnosed with osteoporosis (OP) based on dual energy x-ray absorptiometry (DXA) testing [1], the lifetime risk of a low-impact fracture is 40 % for older women and 13 % for older men [2]. Hip fractures, related to OP, are associated with increased mortality and serious adverse effects on quality of life (QOL) and health care costs [3]. Many older adults know very little about their OP risks or the rationale for screening to identify this silent disease [4, 5]. As suggested by health behavior theories, like the Health Belief Model [6], knowledge of OP, its risks and how to prevent or improve OP, is a first step for patients in making bone health-related behavior changes (i.e. getting adequate amounts of calcium, vitamin D and weight-bearing exercise, taking appropriate pharmacotherapy, preventing falls).

Some randomized controlled trials (RCTs) have demonstrated that educational interventions improve OP-related knowledge [7–16]. Less is known about whether such interventions affect patient satisfaction or QOL [17, 18]. Most of these RCTs examining OP patient-education interventions have been multifaceted or lengthy [7, 12, 13, 15, 16], which are not scalable. To date, only one RCT has examined the effect of an OP education intervention on QOL, reporting a beneficial effect after both three months and one year [16]. To our knowledge only three studies have shown that patients receiving educational or quality improvement interventions were more satisfied with their bone-health care than usual care groups [16, 19, 20]. However, two of these studies were conducted only with older women with an OP diagnosis [16, 19]. The other study only saw improved satisfaction for timeliness in test result notification but not in other measures of satisfaction (e.g. understanding DXA results or treatment options) [20]. Because tailored interventions individualized to the patient’s characteristics are more effective and preferred by patients than standardized interventions [21, 22], we developed a tailored, pragmatic patient-activation intervention.

We reported that 90 % of older adults wanted to receive their DXA results by mail [23]. Therefore, we developed a DXA result letter and a bone-health educational brochure. We then designed and conducted a pragmatic RCT to assess the impact of this intervention on the pathways leading to appropriate pharmacotherapy, health behavior change, and satisfaction with bone-health care, QOL, OP knowledge, and cost-effectiveness (www.ClinicalTrials.gov Identifier NCT01507662). This article describes the effects of the intervention on three patient-reported outcomes: satisfaction with bone health care, QOL, and OP knowledge at 12 and 52 weeks post-baseline. We hypothesized that the intervention would improve bone-health care satisfaction and OP knowledge at both time points, but would not change QOL.

## Methods

### Participants

Patients ≥ 50 years old presenting for DXA between February 2012 and August 2014 at the University of Iowa (UI), University of Alabama at Birmingham (UAB), and Kaiser Permanente of Georgia (KPGA) were invited to participate. We excluded non–English speakers and prisoners. Twenty dollar gift cards were provided after the baseline interview.

### Design and randomization

We used a double-blind, parallel, pragmatic RCT [24]. After patients completed their DXA and baseline interviews they were randomized based on their providers using a computer program written in R [25]. Providers were first ranked within sites based on the number of DXAs they ordered in the previous two years, and then they were randomized within sites using blocks of three into three groups (1:1:1 allocation ratio). Patients of providers in the first group were assigned to the intervention arm, patients of providers in the second group were assigned to the usual care arm, and patients of providers in the third group were randomized to either the usual care or intervention arms (1:1 allocation ratio). We selected three provider randomization groups to assess potential spill-over effects on the main outcomes for usual care patients of providers in the third group.

### Procedures

At baseline, research assistants (RAs) at each site used REDCap™ [26] computer assisted interviewing (CAI) software to interview patients up to four weeks before or three days after their DXA. All KPGA patients and half of the UI patients completed these interviews in person. All UAB patients and the remaining UI patients completed their baseline interviews over the telephone. Three RAs at UI mailed study materials to intervention patients.

### Intervention

Intervention materials included a letter describing results of their DXA (lowest T-score and interpretation [OP, low BMD or normal]), a graphic portrayal of their 10-year probability for a major osteoporotic fracture (using FRAX®; https://www.shef.ac.uk/FRAX/), and a bone-health educational brochure. These materials have been described elsewhere [27, 28].

### Outcomes

Twelve weeks and 52 weeks after their DXA, Iowa Social Science Research Center interviewers telephoned patients at all three sites to conduct follow-up interviews using WinCATI 4 · 2 and 5 · 0 CAI software (Sawtooth Technologies, Northbrook, IL). Interview questions have been described elsewhere [24].

OP Care Satisfaction. This five-item scale assesses patient satisfaction with notification and understanding DXA results, understanding OP treatments, receiving adequate information to make an informed decision, and overall satisfaction with bone-health care. Response options ranged from strongly agree to strongly disagree, with summary scores ranging from 5 (least satisfied) to 25 (most satisfied). Four of these items were used in a previous study [20], with a fifth item related to overall satisfaction added for the current study. Because this was the first use of the satisfaction with OP care scale after its development and initial publication, we used exploratory factor and reliability analyses to explore its psychometric properties. Those results revealed a simple factor structure that was unidimensional with principal factor loadings for each item ranging from 0.53 to 0.77, and an internal consistency reliability (alpha) coefficient of 0.77. At baseline, these items were only asked of patients with prior DXAs because they were irrelevant for DXA naïve patients. All patients were asked these questions at the 12- and 52-week interviews.

Quality of Life. We used three QOL measures. The first was the SF-1 (“In general, would you say your health is excellent, very good, good, fair, or poor”), which was scored as 95, 90, 80, 30, and 15 to reflect the underlying health utilities [29]. The second QOL measure was the EQ-5D-3 L which has five items assessing difficulties with mobility, self-care, activities of daily living, pain, and mood (α = 0 · 70) [30, 31]. Responses (no difficulties, some, or were completely impaired) were converted to health utilities ranging from 0 (death) to 1 (best health state) [30, 31]. The third QOL measure was the EuroQol Visual Analog Scale (VAS) which asks patients to rate their health from 1 (worst health state they can imagine) to 100 (best health state they can imagine) [30, 31].

OP Knowledge. We used the 10-item “Osteoporosis and You” scale [5, 32] to measure. The five responses ranged from strongly agree (SA) to strongly disagree (SD), which were collapsed into “correct” or “incorrect” responses. Correct responses (SA or A for true or SD or D for false statements) were coded “1” with incorrect responses coded “0”. We summed the responses into a total score (α = 0 · 68). We also examined the subscales (biological, lifestyle, consequences, and prevention and treatment).

### Statistical considerations and analysis

PAADRN was powered for guideline concordant treatment as the clinical endpoint. Therefore, for the current analysis, we calculated the statistical power that we would have to detect a standardized effect size of 0.10 with *p* < 0.05 and attrition from baseline as high as 20 %. Those calculations indicated that we would have 91.3 % power to detect such differences. We used multiple imputation techniques to account for missingness (lost to follow-up, patient refused a specific question or responded “don’t know”). We imputed each item separately and constructed the outcomes based on the imputed values. Our primary analysis was based on intention-to-treat (ITT).

We first compared the outcome measures between the intervention and control groups at baseline and at the two follow-ups using t-tests. We then used linear random effects regression methods to adjust for patient clustering within provider and for pre-specified covariates. For patient satisfaction, we first examined differences between intervention and control groups at 12-weeks and 52-weeks (baseline patient satisfaction was only asked for those with prior DXAs). Among those with prior DXAs, we adjusted for baseline satisfaction in separate models. For QOL and OP knowledge we examined differences between baseline and 12-weeks, and baseline and 52-weeks. We used Bonferroni methods to adjust for testing at two time points (12- and 52-weeks) and for using three QOL measures. We examined minimally important differences (MIDs) defined distributionally as improvements ≥ 0.5 standard deviations (SD) [33]. For the EQ-5D utility score we also used an anchor-based approach to predict utility scores for the pairwise SF-1 comparisons (adjusting for age, gender, and race).

We also investigated pre-specified heterogeneity of treatment (HTE) effects. These included median splits on preferred approaches to health care decision-making and treatments [34], those with prior DXAs vs. those without, those on OP-medications at baseline vs. those who were not, those with a history of OP or osteopenia at baseline vs. those who did not, site (UAB vs. KPGA vs. UI), age (<65 vs. 65-75 vs. > 75), men vs. women, Whites vs. non-Whites, education (high school or less vs. some college vs. graduate school), self-rated health (poor vs. fair vs. good vs. very good vs. excellent), having COPD, depression, or prior fracture at baseline (vs. not), FRAX risk (low vs. moderate vs. high), current smoker vs. former smoker vs. never smoked, heavy vs. moderate alcohol consumption, and median splits on weight-bearing exercise.

In sensitivity analyses we used case-wise deletion instead of multiple imputation. Because those results were entirely consistent with the results presented below that used multiple imputation, we only report the latter here. With the Bonferroni adjustments, all *p*-values were 2-tailed with ≤ 0.025 deemed statistically significant for patient satisfaction and OP-related knowledge, and < 0.0083 deemed statistically significant for the three measures of QOL. Analyses were performed using SAS 9 · 4 (SAS Institute Inc., Cary, NC).

## Results

### Participant enrollment and characteristics

**Fig. 1:**
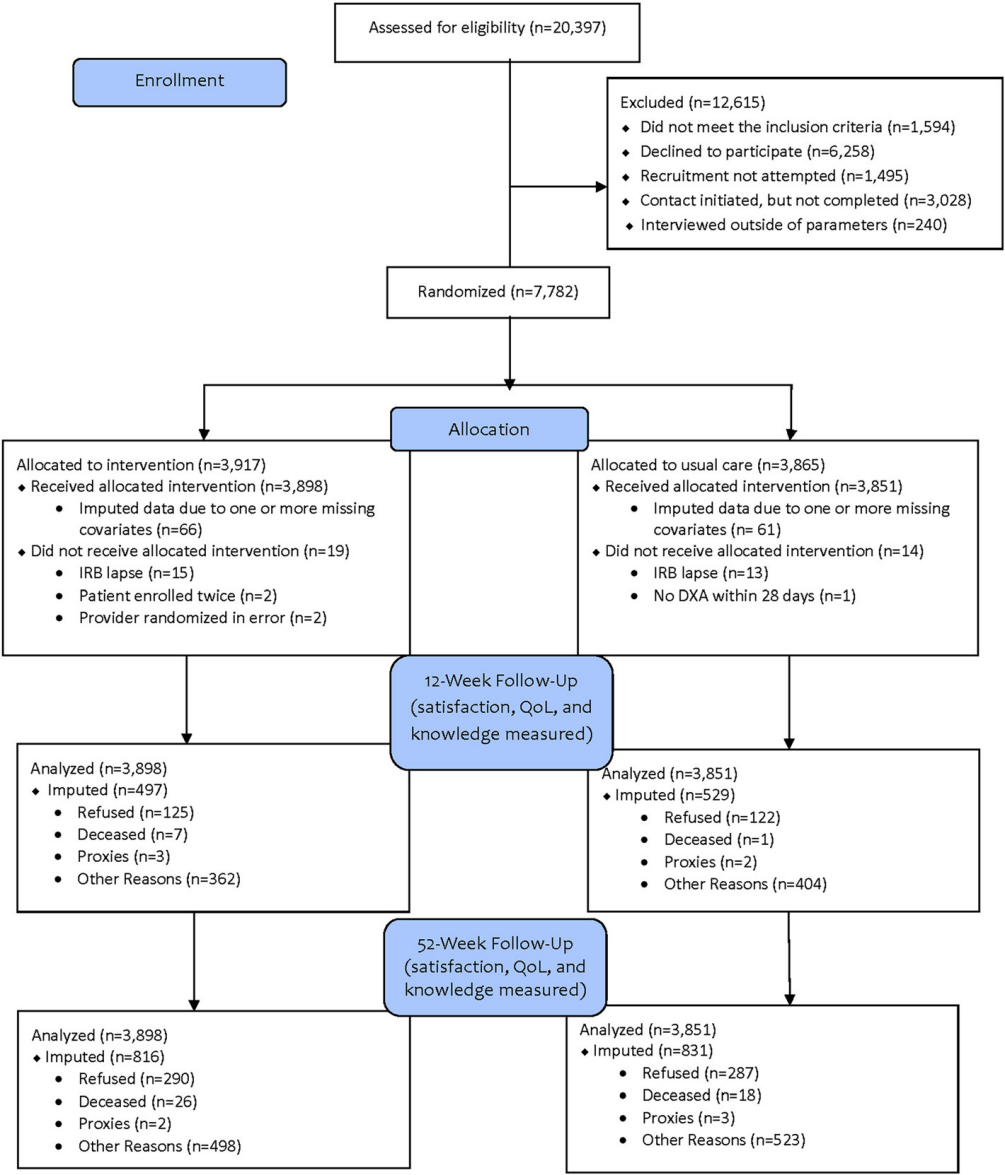
CONSORT Flow Diagram of PAADRN Study

There were 20,397 potentially eligible patients, of whom 7,782 agreed to participate, were interviewed at baseline, and were then randomized to either the intervention or usual care groups (Fig. 1). Of these, 33 patients were randomized in error and were removed from the study, leaving 7,749 patients. Of these, 6,728 (86.8 %) completed the 12-week and 6,107 (78.8 %) completed the 52-week follow-up interviews. All 7,749 randomized participants were included in the analysis using intent-to-treat principles.

Table 1 presents baseline characteristics for all 7,749 participants. The mean age of our participants was 66 years, 84 % were women, 77 % were White, and 67 % had previously undergone DXA. The intervention and usual care groups were similar in age, sex, race, education, and self-reported health. The usual care group, however, was more likely to have had OP prior to baseline (*p* = 0.001), and to have had an index DXA indicating low bone mineral density (BMD) or OP (*p* = 0.001).

**Table 1:** Baseline characteristics by treatment group among the PAADRN participants (*N* = 7,749)

	Intervention (*N* = 3,898)	Control (*N* = 3,851)	*P*-Value
Sociodemographics			
Age, mean (SD)	66.5 (8.4)	66.7 (8.2)	0.246^1^
Women, N (%)	3,259 (83.6)	3,230 (83.9)	0.750^2^
Race/Ethnicity			
White, N (%)	2,981 (76.5)	2,954 (76.7)	0.699^2^
Black, N (%)	842 (21.6)	814 (21.1)	
Other, N (%)	75 (1.9)	83 (2.2)	
Education			
Some high school, N (%)	161 (4.2)	140 (3.7)	0.749^2^
Completed high school, N (%)	819 (21.2)	836 (21.9)	
Some college, N (%)	1,290 (33.4)	1,269 (33.2)	
Completed college, N (%)	785 (20.3)	762 (19.9)	
Graduate school, N (%)	809 (20.9)	814 (21.3)	
Comorbid Conditions			
COPD, N (%)	259 (6.7)	265 (6.9)	0.680^2^
Depression, N (%)	902 (23.2)	885 (23.0)	0.878^2^
Prostate cancer, N (%)	117 (18.3)	88 (14.2)	0.048^2^
Breast cancer, N (%)	416 (10.7)	612 (15.9)	<0.001^2^
Health Habits			
Current smoker, N (%)	295 (7.6)	295 (7.7)	0.873^2^
Past smoker, N (%)	1,478 (37.9)	1,388 (36.1)	0.095^2^
Current alcohol user, N (%)	1,768 (45.4)	1,808 (47.0)	0.157^2^
Self-reported Health Status			
Excellent, N (%)	445 (11.4)	494 (12.8)	0.329^2^
Very Good, N (%)	1,443 (37.1)	1,373 (35.7)	
Good, N (%)	1,280 (32.9)	1,253 (32.6)	
Fair, N (%)	571 (14.7)	566 (14.7)	
Poor, N (%)	150 (3.9)	159 (4.1)	
Bone Health			
Prior DXA, N (%)	2,606 (66.9)	2,590 (67.3)	0.719^2^
History of OP, N (%)	794 (20.6)	909 (23.8)	0.001^2^
History of OP treatment, N (%)	1,438 (36.9)	1,502 (39.0)	0.055^2^
Glucocorticoids Use, N (%)	593 (15.2)	576 (15.0)	0.753^2^
Study DXA Results			
Normal, N (%)	1,133 (29.1)	990 (25.7)	0.001^2^
Low BMD, N (%)	2,052 (52.6)	2,066 (53.6)	
Osteoporosis, N (%)	713 (18.3)	795 (20.6)	
Lowest T-Score, mean (SD)	-1.62 (1.1)	-1.55 (1.1)	0.002^1^
10-year Fracture Risk (FRAX), mean (SD)	12.0 (9.2)	12.3 (9.1)	0.101^1^

^1^ *P*-value from Two-sample T-Test

^2^ *P*-value is from Pearson Chi-square Test

### Patient satisfaction with OP health care

Intervention patients had significantly greater (better) levels of patient satisfaction with their bone-health care than the usual care group at both 12-weeks (1.0 points, standardized effect size = 0.28, *p* < 0.001; Table 2) and 52-weeks (0.6 points, standardized effect size = 0.21, *p* < 0.001). Adjustments for clustering within providers and the covariates did not alter these differences (1.02 points at 12-weeks and 0.63 at 52-weeks, *p* < 0.0005; Table 3). Patients in the intervention group had 58 % greater odds of having an MID improvement (AOR = 1.58, *p* < 0.0005) at 12-weeks and 34 % greater odds at 52-weeks (AOR = 1.34, *p* < 0.005). We observed comparable results in all of the HTE comparison groups (not shown).

**Table 2:** Unadjusted means (SDs) on all 7,749 PAADRN participants at baseline, 12- and 52-weeks using intention-to-treat (ITT)

	Baseline			12-weeks			52-weeks		
	Intervention	Control	*P*-value	Intervention	Control	*P*-value	Intervention	Control	*P*-value
OP care satisfaction (5-25)	18.9 (2.7)^a^	19.1 (2.8)^a^	0.011	21.1 (3.2)	20.1 (4.0)	<0.001	21.1 (3.2)	20.5 (3.8)	<0.001
Quality of life									
SF-1 (15/30/80/90/95; poor to excellent)	75.2 (23.8)	75.1 (24.1)	0.925	74.6 (24.2)	74.8 (24.4)	0.746	74.5 (24.1)	75.4 (23.8)	0.132
EuroQol EQ5D-3 L utility score (0-1)	0.8 (0.2)	0.8 (0.2)	0.676	0.8 (0.2)	0.8 (0.2)	0.547	0.8 (0.2)	0.8 (0.2)	0.72
EuroQol visual analog scale of Euroqol (0-100)	78.1 (16.7)	78.3 (16.7)	0.590	77.5 (17.6)	78.2 (17.2)	0.083	77.7 (17.6)	78.2 (17.1)	0.244
OPc knowledge – scale score (0-10)	7.5 (1.9)	7.5 (1.8)	0.712	7.8 (1.6)	7.8 (1.6)	0.759	7.8 (1.6)	7.8 (1.6)	0.476
Biological risk factors	2.3 (0.9)	2.3 (0.9)	0.345	2.5 (0.8)	2.4 (0.8)	0.495	2.5 (0.8)	2.5 (0.8)	0.724
Lifestyle risk factors	1.8 (0.4)	1.8 (0.4)	0.807	1.9 (0.3)	1.9 (0.4)	0.089	1.9 (0.4)	1.9 (0.4)	0.327
Consequences	1.7 (0.6)	1.7 (0.6)	0.688	1.7 (0.5)	1.7 (0.5)	0.471	1.7 (0.5)	1.7 (0.5)	0.485
Prevention and treatment	1 (0.8)	1 (0.8)	0.974	1 (0.8)	1 (0.8)	0.726	0.9 (0.8)	0.9 (0.8)	0.832

^a^These are means and standard deviations among those who had a DXA prior to baseline interview

**Table 3:** Regression coefficients for the intervention on satisfaction with OP care, QOL, and OP knowledge from the intention-to-treat (ITT) random effects models (*N* = 7,749)

		12-weeks		52-weeks	
		Crude	Adjusted	Crude	Adjusted
OP care satisfaction (5-25)^a^	Estimate	1.02*	1.02*	0.61*	0.62*
	95 % CI	( 0.82, 1.22)	( 0.83, 1.22)	( 0.40, 0.82)	( 0.43, 0.82)
Quality of life					
SF-1 (15/30/80/90/95; poor to excellent)	Estimate	0.21	-0.05	-0.37	-0.66
	95 % CI	( -0.74, 1.16)	( -0.93, 0.83)	( -1.33, 0.58)	( -1.52, 0.21)
EuroQol EQ5D-3 L, utility score (0-1)	Estimate	0.00	0.00	0.00	0.00
	95 % CI	( -0.01, 0.01)	( -0.01, 0.00)	( -0.01, 0.01)	( -0.01, 0.01)
EuroQol visual analog scale (0-100)	Estimate	-0.44	-0.59	-0.42	-0.47
	95 % CI	( -1.13, 0.26)	( -1.23, 0.05)	( -1.18, 0.33)	( -1.18, 0.24)
OP knowledge scale score (0-10)	Estimate	0.01	0.01	0.04	0.03
	95 % CI	( -0.07, 0.1)	( -0.06, 0.08)	( -0.03, 0.12)	( -0.04, 0.09)

*p < 0.0005

^a^These are models on outcomes at 12-weeks and 52-weeks without adjustment for baseline values

Additionally, we repeated the analyses at the item level. In the pooled unadjusted and adjusted analyses at both 12- and 52–weeks, the intervention group reported significantly (*p* ≤ 0.002) higher satisfaction for each of the five satisfaction items (data not shown). When stratified by prior DXA use, the unadjusted results were significant (*p* ≤ 0.03) for each of the five satisfaction items with one exception (data not shown); at 52-weeks among prior DXA users the intervention did not have a significant effect (*p* = 0.14) on the item related to their overall-satisfaction with bone-health care.

### Quality of life

Intervention and control participants had similar mean scores on all three QOL measures at baseline (SF-1, *p* = 0.925; EQ-5D-3 L, *p* = 0.676; and, EuroQol VAS, *p* = 0.590; Table 2). Mean scores for all three QOL measures did not differ between groups at either 12- or 52-weeks (Table 2). The changes from baseline to the two follow-ups are not significant for all three QOL measures (data not shown). No significant differences were observed in the random effects models, either, for any of the three QOL measures (Table 3). Similarly, no significant differences were observed for MID improvements for any of the three QOL measures. In additional analyses at the item level, no significant (*p* > 0.05) effects of the intervention were observed for any of the QOL items (data not shown).

### OP Knowledge

The intervention and usual care groups had the same mean scores for OP knowledge (7.5, SD 1.9; Table 2). OP knowledge increased significantly by 0.3 points for both the intervention and usual care groups between baseline and the 12- and 52-week follow-ups (*p* < 0.001), but there was no difference in the amount of the increase between the two groups (*p* =0.759 at 12-weeks and *p* = 0.479 at 52-weeks; Table 2). Adjustment for patient clustering within provider, and for the covariates did not alter these findings (Table 3). In additional analyses at the item level, no significant (*p* > 0.05) effects of the intervention were observed for any of the OP knowledge items (data not shown).

## Discussion

There is growing interest in engaging patients in their own healthcare [35, 36]. Tailoring health communication to be more patient-centered is becoming more common. Yet, it is unclear whether greater access to tailored, DXA testing communication results in any measurable improvements in patient reported outcomes. We designed a, pragmatic, multi-site, RCT to evaluate the effects of a tailored DXA result letter accompanied by a bone-health educational brochure on patient satisfaction with OP care, QOL, and OP knowledge. Our results revealed significantly improved patient satisfaction with OP care in the intervention group compared to the usual care group. Intervention patients had 58 % greater odds of improving by at least an MID (0.5 SD) at 12-weeks (*p* < 0.0005) and 34 % greater odds off improving by at least an MID at 52-weeks (*p* < 0.005). However, we found no differences in terms of QOL or OP knowledge between the intervention and usual care groups.

These findings are important because, to our knowledge, this is the first positive study to include a comparison group, and to include patients with and without OP. Patient satisfaction is recognized as an important dimension of healthcare quality. Medicare now evaluates patient satisfaction [37] which will soon be used in determining preventive service reimbursements for doctors and hospitals. The intervention materials were pilot tested to ensure comprehension as well as patient preferences for information and design [27, 28]. Tailoring test result communications to patients may improve their satisfaction with other types of testing as well.

As important as the effect on satisfaction with OP care is, failure to improve QOL or OP knowledge in our study also must be considered. We did hypothesize that the patient-activation intervention would not affect overall QOL because OP care is a small component of the healthcare received by older adults who may have several comorbidities. Indeed, OP would likely have minimal effects on QOL in the short-term until a fracture occurs, at which point profound effects on QOL. Thus, our results are consistent with prior studies of OP patient education interventions on QOL [11, 38], in which only one reported a significant improvement among Malaysian women taking bisphosphonates [16]. Moreover, trials of OP therapies, which demonstrate reduced fracture rates, seldom are powered to detect an effect on QOL for some of the reasons noted.

The absence of an effect of the patient-activation intervention on OP knowledge is surprising and contrary to our expectations. Prior OP-education interventions have reported significant improvements in knowledge [7–16]. In contrast, we found that OP knowledge significantly increased in both intervention and usual care groups, but that the magnitude of these improvements was the same. This may be due to the measure of OP knowledge. First, the reliability of the OP knowledge measure was only marginally acceptable (α = 0.68) [39]. Second, this was the first RCT to use the “Osteoporosis and You” measure, and the two prior observational studies assessing its psychometric properties included younger women and not men [5, 32]. Third, significant practice effects (about half of an additional correct answer) were observed and these may have created a ceiling effect that constrained our ability to detect short-term differences. Finally, the null effect may be due to the fact that neither the patient-activation letter nor the educational brochure were targeted to the “Osteoporosis and You” measure, although an ad hoc analysis of the four items most closely reflecting the intervention did not reveal an effect either (data not shown). Although several other measures of OP knowledge were available when our study began, we eliminated them because they were too long and cumbersome [40, 41] or were designed for younger women who did not have OP [42]. Improved measures of OP knowledge are needed, particularly among those known to have OP.

Despite its strengths, our RCT had limitations. First, the patient satisfaction with OP health care scale had not been used in RCTs designed to improve bone health. Second, we did not use an OP-specific QOL measure, which might have been more responsive to our patient-activation intervention. Lastly, given the clinical centers used, our study population may not have been representative of all osteoporosis patients.

## Conclusion

In conclusion, because of increases in the number and percentage of older Americans at risk for OP and hip fractures, there is a growing need for better OP health care and patient knowledge about the prevention, treatment, and consequences of this disease that remains silent until fracture. We developed a pragmatic and tailored patient-activation intervention that improved OP care satisfaction. Future research and quality improvement projects should examine whether patient satisfaction scores in other clinical domains or in general would increase when providing patients with their test results in a tailored manner.

## Abbreviations

BMD: Bone mineral density
CAI: Computer assisted interviewing
DXA: Dual energy x-ray absorptiometry
HTE: Heterogeneity of treatment
ITT: Intention-to-treat
KPGA: Kaiser permanente of Georgia
OP: Osteoporosis
PAADRN: Patient activation after DXA result notification
QOL: Quality of life
RAs: Research assistants
RCT: Randomized controlled trial
SA: Strongly agree
SD: Standard deviation
SD: Strongly disagree
UAB: University of Alabama at Birmingham
UI: University of Iowa
VAS: EuroQol visual analog scale
